# CT-based artificial intelligence system complementing deep learning model and radiologist for liver fibrosis staging

**DOI:** 10.1016/j.isci.2025.112224

**Published:** 2025-03-17

**Authors:** Shuang Zheng, Wenao Ma, Lin Mu, Kan He, Jianfeng Cao, Tiffany Y. So, Lei Zhang, Mingyang Li, Yanan Zhai, Feng Liu, Shunlin Guo, Longlin Yin, Liming Zhao, Lei Wang, Heather H.C. Lee, Wei Jiang, Junqi Niu, Pujun Gao, Qi Dou, Huimao Zhang

**Affiliations:** 1 Department of Radiology, The First Hospital of Jilin University, Changchun, China; 2 Department of Computer Science and Engineering, The Chinese University of Hong Kong, Hong Kong, China; 3 Department of Imaging and Interventional Radiology, Prince of Wales Hospital, Hong Kong, China; 4 Department of Radiology, The First Hospital of Lanzhou University, The First Clinical Medical College of Lanzhou University, Intelligent Imaging Medical Engineering Research Center of Gansu Province, Radiological Clinical Medicine Research Center of Gansu Province, Lanzhou, China; 5 The First Clinical Medical College of Lanzhou University, Lanzhou, China; 6 Department of Radiology, Sichuan Academy of Medical Sciences & Sichuan Provincial People’s Hospital, Chengdu, China; 7 Department of Diagnostic Radiology, Princess Margaret Hospital, Hong Kong, China; 8 National Health Commission Capacity Building and Continuing Education Center, Department of Big Data, Beijing, China; 9 Department of Hepatology, The First Hospital of Jilin University, Changchun, China

**Keywords:** Public health, Pathophysiology, Artificial intelligence

## Abstract

Noninvasive methods for liver fibrosis staging are urgently needed due to its significance in predicting significant morbidity and mortality. In this study, we developed an automated DL-based segmentation and classification model (Model-C). Test-time adaptation was used to address data distribution shifts. We then established a deep learning-radiologist complementarity decision system (DRCDS) via a decision model determining whether to adopt Model-C’s diagnosis or defer to radiologists. Model-C (AUCs of 0.89–0.92) outperformed models based on liver (AUCs: 0.84–0.90) or spleen (AUCs: 0.69–0.70). With test-time adaptation, the Obuchowski index values of Model-C in three external sets improved from 0.81, 0.73, and 0.73 to 0.85, 0.85, and 0.81. DRCDS performed slightly better than Model-C or senior radiologists, with 73.7%–92.0% of cases adopting Model-C’s diagnosis. In conclusion, DRCDS could diagnose liver fibrosis with high accuracy. Additionally, we provided solutions to model generalization and human-machine complementarity issues in multi-classification problems.

## Highlights

• DRCDS improved DL model’s essential design and complemented it with radiologists
• DRCDS enhanced DL model’s or radiologists’ efficiency for liver fibrosis staging
• Test-time adaptation could be used to enhance model’s generalization capability

## Graphical abstract

**Figure:**
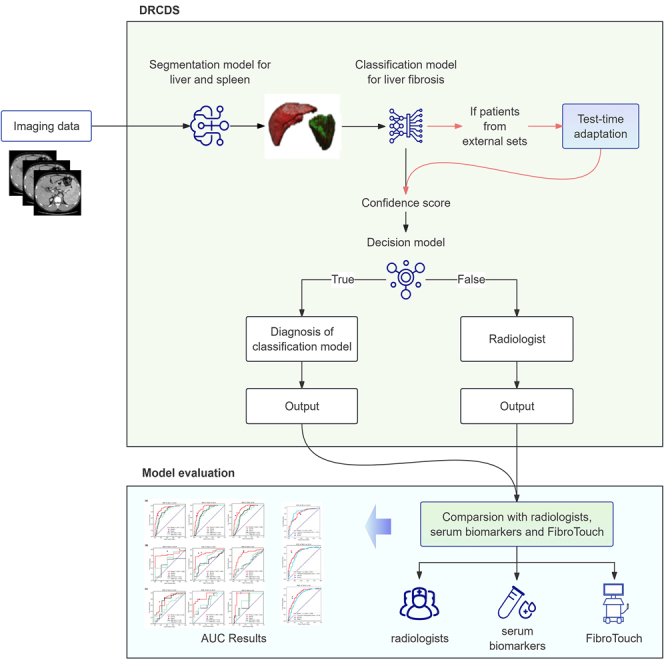

## Introduction

Liver fibrosis staging is important for monitoring disease progression, establishing prognosis and making treatment decisions in chronic liver disease.^1^^,^^2^^,^^3^ Although liver biopsy is the current reference standard, its high cost and related complications limit its utility in routine monitoring and screening.^4^ Therefore, the development of noninvasive tools (NITs) remains a long-standing unmet clinical need.

Commonly used NITs for liver fibrosis staging include simple serum markers (aspartate transaminase-to-platelet ratio [APRI] and fibrosis-4 index [FIB-4]) and transient elastography (TE). However, these NITs lack robust diagnostic accuracy in patients with inflammatory activity. The TE examination also requires specialized equipment and expertise, and its measurement results are dependent on the operator. Morphologic changes could suggest the presence of chronic liver disease or cirrhosis, including blunted liver edges, widened liver fissures, coarse hepatic texture, liver surface nodularity, liver segmental volume redistribution, and portal hypertension (i.e., ascites, splenomegaly, or varices).^5^ Nevertheless, visual assessment is subjective, and the sensitivity of these findings is limited during the early stages of fibrosis.

Deep learning (DL) has facilitated substantial progress in medical image analysis, leading to the development of promising automated DL models for liver fibrosis staging.^6^^,^^7^^,^^8^^,^^9^^,^^10^ Among them, Choi et al. presented an automated liver segmentation and classification DL system using a large multi-center portal venous CT dataset and achieved an AUC of 0.96, 0.97 and 0.95 for significant fibrosis, advanced fibrosis and cirrhosis.^9^ Hectors et al. proposed a DL algorithm consisting of automatic mid-liver slices selection, segmentation and fibrosis stages prediction based on gadoxetic acid-enhanced hepatobiliary phase MRI, with the AUCs of 0.91, 0.90, and 0.85 separately.^8^ However, those studies primarily focused on liver features, disregarding the essential role of portal hypertension indicators in liver fibrosis diagnosis, which limits model’s diagnostic performance. Moreover, no study has tried to address the domain shift issues existing in the real world, undermining the generalization capability of DL models. Additionally, none of those studies probed into the strategies for effectively integrating DL models with human decision-makers.

Because of the wide availability and standardization of CT and its frequent use for severity assessment and hepatocellular carcinoma screening in chronic liver disease, we developed an automated deep learning radiologist complementarity decision system (DRCDS), a system for liver fibrosis staging using portal venous CT, by first combing both liver and spleen features to improve DL model’s diagnostic performance and then improving its generalizability and complementing it with radiologist to make it more suitable for clinical application. We further validated it across multiple centers and compared its performance with that of serum markers and TE.

## Results

### Study design

**Figure 1:**
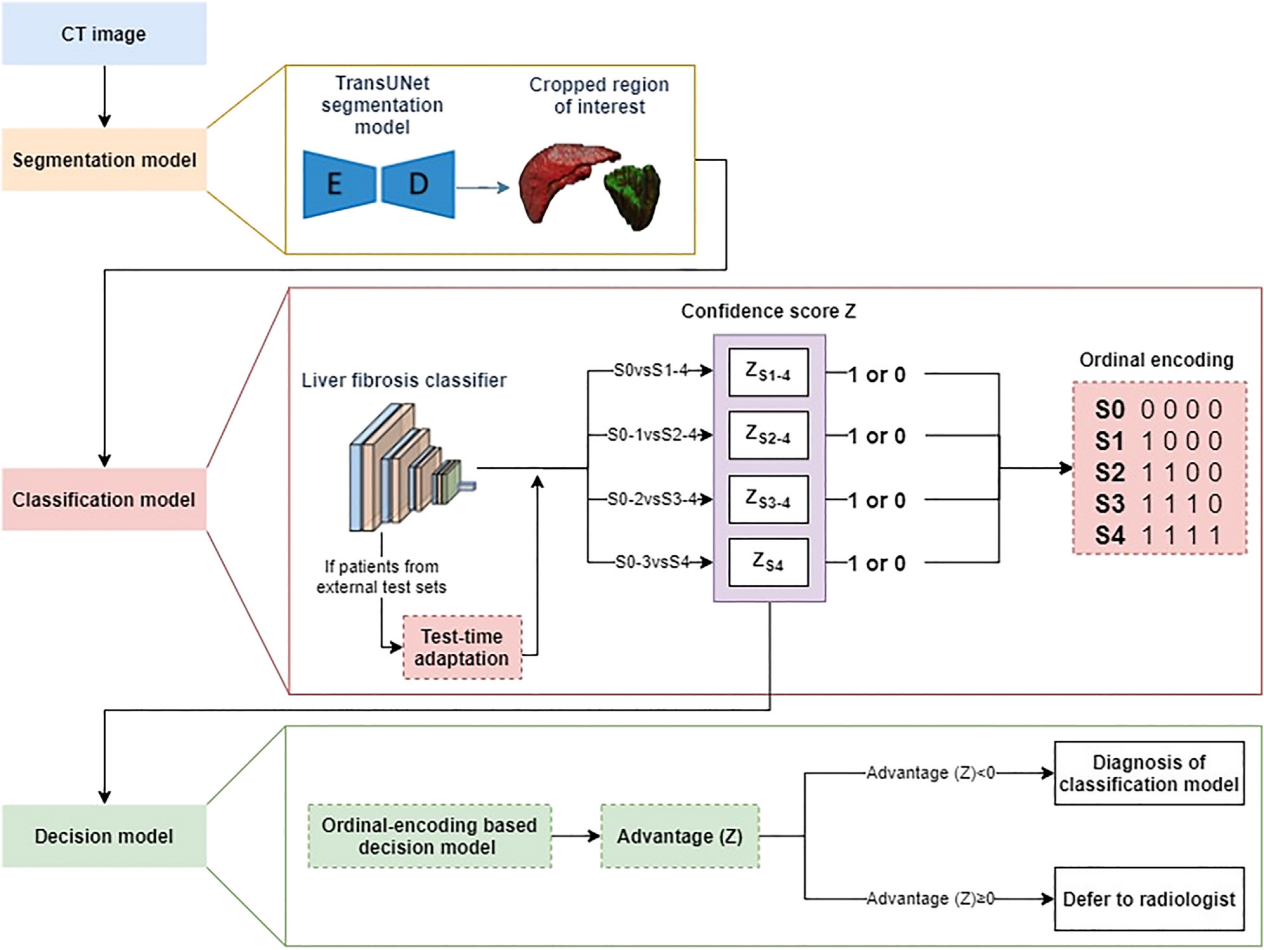
Flowchart of deep learning-radiologist complementary decision model (DRCDS) The DRCDS consists of a cascade of three modules: a segmentation model for liver and spleen, a classification model for liver fibrosis staging, and a decision model for the complementation with classification model and radiologist. The automatically segmented liver and spleen were forwarded to the liver fibrosis classifier, and the five classes output are converted into 4-bit ordinal vectors through ordinal encoding. For cases from unseen external test sets, a test-time adaptation scheme was used to address data distribution shift. The confidence score outputted by each classification task is then forwarded into decision model and compute the value of advantage (z). If advantage (z)≥0, the system decides to defer to the radiologist. Otherwise, the decision model decides to use the output of the classification model and radiologist’s read can be saved.

The DRCDS consisted of three sequential modules: a DL-based automated liver and spleen segmentation model, liver fibrosis classification model and a decision model, as shown in Figure 1. To train the segmentation model more effectively, we adopted a human-in-the-loop strategy. We also improved the essential designs of classification model by combining liver and spleen, referring to radiologists’ experience. To improve classification model’s generalizability, we used a test-time learning algorithm. Further, to complement the strengths of DL model and radiologists, we introduced an ordinal encoding-based decision model, which utilizing the confidence score generated by DL model to determine whether to trust the output of DL model or defer the case to radiologists. Finally, we compared the performance of DRCDS with that of serum markers and TE.

### Multi-center data characteristics

**Figure 2:**
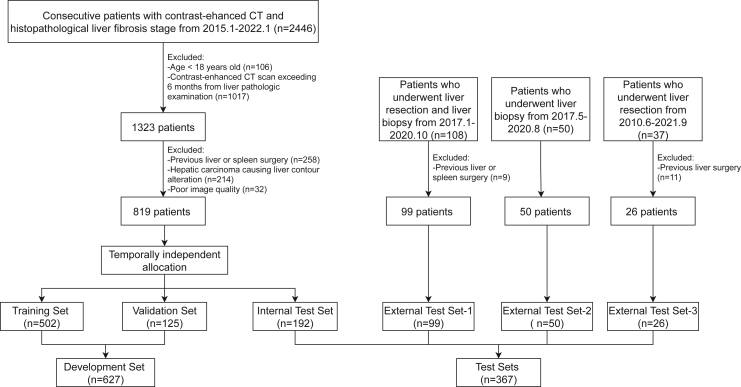
Flowchart for patient selection in the study and breakdown of training, validation, and test sets

Figure 2 illustrates the patient selection flowchart. The characteristics of the multi-center dataset (*n* = 994) are outlined in Table 1. The 819 patients in the Changchun dataset (mean age, 53.2 ± 11.5 years [SD]; 546 men) were split into training (*n* = 502), validation (*n* = 125), and internal test sets (*n* = 192). The three external test sets consisted of 175 patients (mean age, 53.9 ± 12.1 years [SD]; 91 men) from Lanzhou (external test set-1, *n* = 99), Chengdu (external test set-2, *n* = 50), and Hong Kong (external test set-3, *n* = 26).

**Table 1:** Patient characteristics of the training, validation and test sets

Characteristic	Training Set	Validation Set	Internal Test Set	External Test Set-1	External Test Set-2	External Test Set-3
No. of patients	502	125	192	99	50	26
No. of men (%)	343 (68.3)	86 (68.8)	117 (60.9)	46 (46.7)	23 (46.0)	22 (84.6)
Age(y)/mean ± SD	53.5 ± 11.3	52.6 ± 12.5	52.4 ± 11.6	51.7 ± 12.6	50.9 ± 14.4	68 ± 8.11
Underlying liver disease/No. (%)						
Hepatitis B	291 (58.0)	67 (53.6)	86 (44.8)	33 (33.3)	7 (14.0)	12 (46.2)
Hepatitis C	46 (9.2)	12 (9.6)	13 (6.8)	2 (2.0)	0 (0.0)	8 (30.8)
Autoimmune^a^	12 (2.4)	7 (5.6)	8 (4.2)	18 (18.2)	20 (40.0)	0 (0.0)
Others^b^	76 (15.1)	20 (16.0)	24 (12.5)	45 (45.5)	23 (46.0)	5 (19.2)
None	77 (15.3)	19 (15.2)	61 (32.3)	1 (1.0)	0 (0.0)	1 (3.8)
Hepatic tumor/No. (%)						
Hepatocellular carcinoma	352 (70.1)	82 (65.6)	99 (51.6)	1 (1.0)	0 (0.0)	20 (77.0)
Metastatic tumors	13 (2.6)	2 (1.6)	14 (7.3)	0 (0.0)	0 (0.0)	0 (0.0)
Benign tumors^c^	32 (6.4)	9 (7.2)	35 (18.2)	3 (3.0)	0 (0.0)	1 (3.8)
None	105 (20.9)	32 (25.6)	44 (22.9)	95 (96.0)	50 (100.0)	5 (19.2)
Pathologic confirmation/No. (%)						
US-guided biopsy	87 (17.3)	23 (18.4)	30 (15.6)	67 (67.7)	50 (100.0)	1 (3.8)
Resection	415 (82.7)	102 (81.6)	162 (84.4)	32 (32.3)	0 (0)	25 (96.2)
Histologic grade/No. (%)						
S0	96 (19.1)	25 (20.0)	71 (37.0)	2 (2.0)	2 (4.0)	1 (3.8)
S1	64 (12.7)	17 (13.6)	20 (10.4)	3 (3.0)	17 (34.0)	0 (0.0)
S2	62 (12.4)	13 (10.4)	22 (11.5)	16 (16.2)	17 (34.0)	3 (11.5)
S3	31 (6.2)	7 (5.6)	15 (7.8)	26 (26.3)	9 (18.0)	2 (7.7)
S4	249 (49.6)	63 (50.4)	64 (33.3)	52 (52.5)	5 (10.0)	20 (77.0)

a Including autoimmune hepatitis, autoimmune cholangitis, primary biliary cirrhosis and primary sclerosing cholangitis.

b Including fatty liver disease, Wilson disease, toxic hepatitis and unknown liver disease.

c Including cavernous hemangioma, hepatic adenoma and focal nodular hyperplasia.

### Evaluation of DRCDS

#### Performance of DL-based automated segmentation algorithm for liver and spleen

The segmentation model was highly accurate and generalizable to external test sets, Dice results for liver and spleen segmentation achieved 97.59% and 96.82% (internal test set), 96.61% and 95.74% (external test set-1), 96.10% and 95.25% (external test set-2) and 93.22% and 94.24% (external test set-3). The efficiency of human-in-the-loop strategy was detailed in Figure S1.

#### Performance of DL-based classification models for liver fibrosis and comparison with radiologists

**Figure 3:**
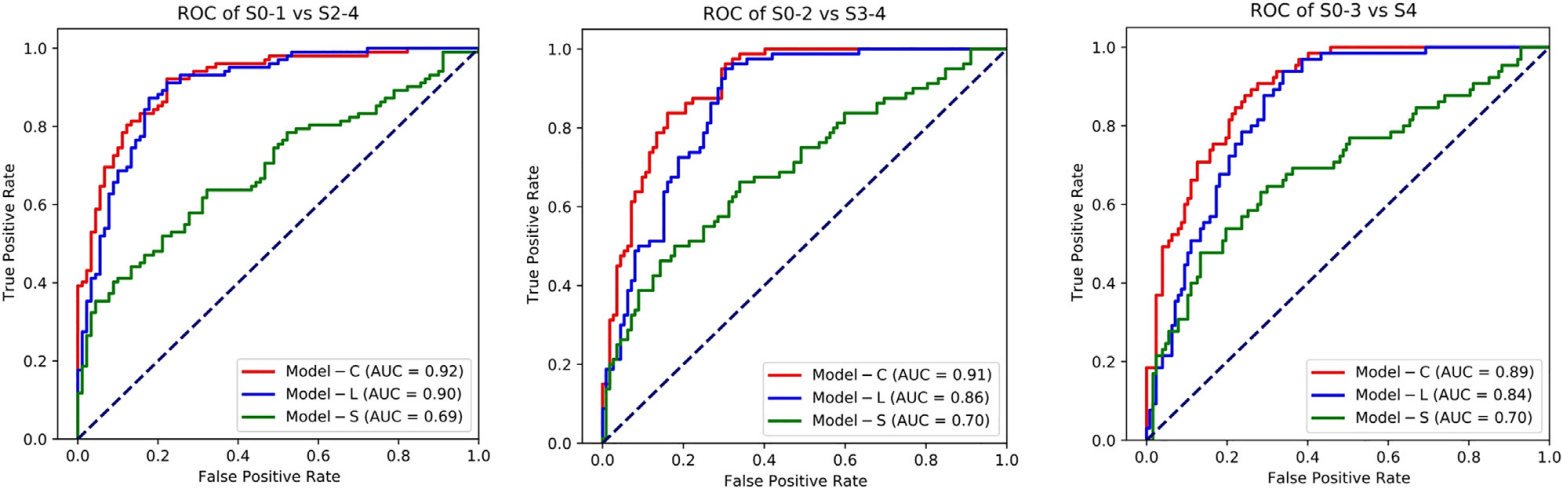
Receiver operating characteristic curves of Model-L, Model-S, and Model-C for diagnosis of significant fibrosis, advanced fibrosis and cirrhosis on internal test set

We first validated Model-C (liver and spleen), Model-L (only liver), and Model-S (only spleen), and compared their performance using the internal test set. Model-C had the best performance, with AUCs for diagnosing significant fibrosis (S2–4), advanced fibrosis (S3–4), and cirrhosis (S4) of 0.92 (95%CI:0.88–0.96), 0.91 (95%CI:0.87–0.95), and 0.89 (95%CI:0.85–0.94) respectively, outperforming Model-L (AUCs:0.90, 0.86, and 0.84 for S2–4, S3–4, and S4 with *p* = 0.31, 0.01, and 0.01, respectively) and Model-S (AUCs:0.69, 0.70, and 0.70 for S2–4, S3–4, and S4, respectively, with *p* < 0.01 for all comparisons). Similarly, the Obuchowski index value of Model-C (0.90, 95%CI: 0.87–0.92) was higher than that of Model-L (0.84, *p* = 0.01) and Model-S (0.70, *p* < 0.01) (Figure 3; Table S1). Stratification analysis revealed no significant differences (*p* > 0.05 for all comparisons) in the Obuchowski index values of Model-C across different subgroups based on patient characteristics (including gender and age), inflammation condition, and CT acquisition data (Table S2).

Subsequently, Model-C was validated using the external test sets and test-time adaptation was employed to improve generalization. Without test-time adaptation, the Obuchowski index values of Model-C in the External Test Sets 1–3 (0.81, 0.73, and 0.73, respectively) noticeably dropped compared to its performance on the internal test set, demonstrating a distribution shift issue. After applying test-time adaptation, the Obuchowski index values improved to 0.85, 0.85, and 0.81, respectively. Moreover, other evaluation metrics such as AUC, sensitivity, and specificity consistently increased (Table 2).

**Table 2:** Diagnostic performance of Model-C on external test sets with and without test-time adaptation

	Without test-time adaptation	Without test-time adaptation	Without test-time adaptation	With test-time adaptation	With test-time adaptation	With test-time adaptation
	External Test Set-1	External Test Set-2	External Test Set-3	External Test Set-1	External Test Set-2	External Test Set-3
Significant Fibrosis (S2-4)						
AUC	0.80 (0.70, 0.91)	0.62 (0.45, 0.79)	0.74 (0.58, 0.89)	0.86 (0.77, 0.94)	0.79 (0.68, 0.88)	0.80 (0.65, 0.95)
Sensitivity	0.79 (0.71, 0.87) [74/94]	0.65 (0.45, 0.85) [20/31]	0.96 (0.92, 0.99) [24/25]	0.95 (0.90. 0.99) [89/94]	0.84 (0.71, 0.97) [26/31]	0.92 (0.84, 0.99) [23/25]
Specificity	0.20 (0.00, 0.40) [1/5]	0.53 (0.30, 0.75) [10/19]	0.00 (0.00, 0.00) [0/1]	0.20 (0.00, 0.40) [1/5]	0.74 (0.54, 0.93) [14/19]	0.00 (0.00, 0.00) [0/1]
Advanced Fibrosis (S3-4)						
AUC	0.83 (0.73, 0.92)	0.72 (0.57, 0.87)	0.76 (0.56, 0.96)	0.88 (0.81, 0.96)	0.90 (0.83, 0.97)^a^	0.81 (0.63, 0.98)
Sensitivity	0.85 (0.77, 0.93) [66/78]	0.71 (0.57, 0.85) [10/14]	0.77 (0.61, 0.93) [17/22]	0.87 (0.80, 0.95) [68/78]	0.79 (0.57, 1.00) [11/14]	0.82 (0.66, 0.98) [18/22]
Specificity	0.62 (0.41, 0.83) [13/21]	0.75 (0.61, 0.89) [27/36]	0.25 (0.01, 0.51) [1/4]	0.62 (0.41, 0.83) [13/21]	0.81 (0.68, 0.93) [29/36]	0.5 (0.01, 0.99) [2/4]
Cirrhosis (S4)						
AUC	0.81 (0.73, 0.90)	0.80 (0.61, 0.99)	0.68 (0.46, 0.90)	0.84 (0.76, 0.92)	0.92 (0.85, 0.99)	0.71 (0.50, 0.92)
Sensitivity	0.83 (0.75, 0.92) [43/52]	1.00 (1.00, 1.00) [5/5]	0.75 (0.56, 0.94) [15/20]	0.88 (0.80, 0.97) [46/52]	1.0 (1.00, 1.00) [5/5]	0.75 (0.56, 0.94) [15/20]
Specificity	0.68 (0.55, 0.81) [32/47]	0.80 (0.68, 0.92) [36/45]	0.33 (0.01, 0.71) [2/6]	0.68 (0.55, 0.81) [32/47]	0.82 (0.71, 0.93) [37/45]	0.5 (0.01, 0.90) [3/6]
Obuchowski index	0.81 (0.78, 0.84)	0.73 (0.70, 0.76)	0.73 (0.64, 0.82)	0.85 (0.82, 0.88)	0.85 (0.82, 0.88)^a^	0.81 (0.72, 0.90)

Data in parentheses are 95% confidence intervals.

AUC, area under the receiver operating characteristic curve.

a Significantly different from the results of Model-C without test-time adaptation (*p* < 0.05). Mann-Whitney U test was performed.

The Obuchowski index values of radiologists reports were 0.91, 0.87, and 0.84 for the internal test set and external test sets-1 and -2, which were similar to that of Model-C (*p* = 0.54, 0.28, and 0.64, respectively). In the Internal Test Set, when sensitivity was fixed, the reports had higher specificity when diagnosing cirrhosis (0.93 vs. 0.89), whereas Model-C demonstrated higher specificity when diagnosing significant fibrosis (0.90 vs. 0.93). Similarly, when specificity was fixed, the reports exhibited higher sensitivity when diagnosing cirrhosis (0.66 vs. 0.52), whereas Model-C demonstrated higher sensitivity when diagnosing significant fibrosis (0.67 vs. 0.72). However, this trend was not observed in external test sets-1 and -2. Reports were not available in external test set-3.

#### Performance of deep learning—radiologist complementarity decision system

**Figure 4:**
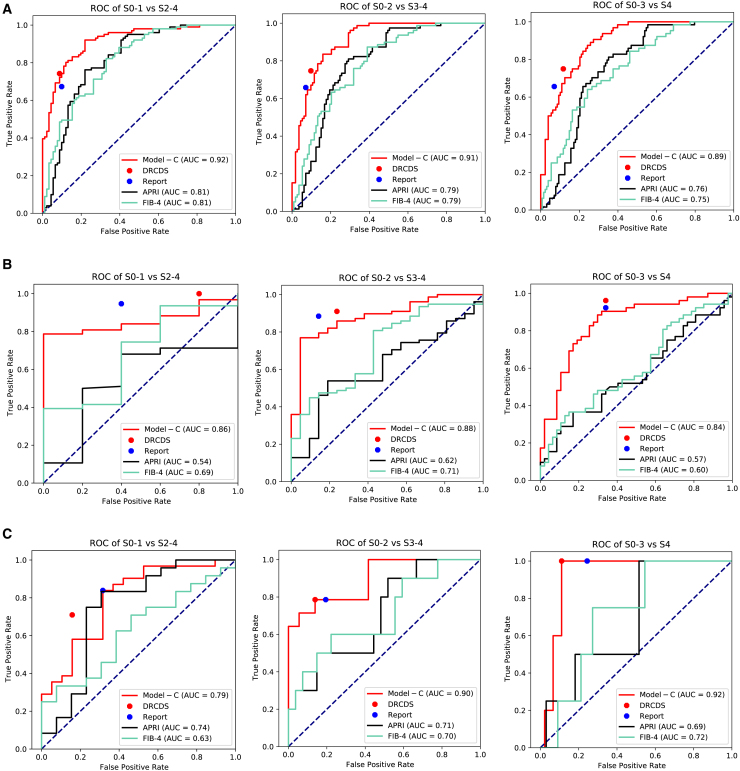
Performance of DRCDS compared to that of Model-C, reports and serum fibrosis test (A) Internal test sets, (B) External test set-1, (C) External test set-2. APRI = aspartate transaminase-to-platelet ratio index. DRCDS = deep learning—radiologist complementarity decision system. FIB-4 = fibrosis-4 index.

**Figure 5:**
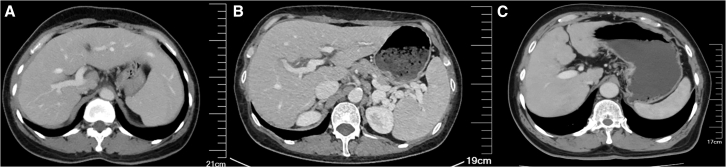
Examples for which the DRCDS provided the correct diagnosis (A) A 50-year-old woman with hepatitis B and pathologic fibrosis stage of S4. DRCDS deemed Model-C (S2) unreliable and diagnosed correctly by deferring to radiologist. The radiologists’ visual assessment of liver fibrosis was S4. (B) A 56-year-old woman with primary biliary cirrhosis and pathologic fibrosis stage of S2. DRCDS deemed Model-C (S2) reliable and diagnosed correctly, with radiologists’ read saved. The radiologists’ visual assessment of liver fibrosis was S4. (C) A 62-year-old man with hepatitis B and pathologic fibrosis stage of S4. DRCDS deemed Model-C (S4) reliable and diagnosed correctly, with radiologists’ read saved. The radiologists’ visual assessment of liver fibrosis was S4. DRCDS = deep learning—radiologist complementarity decision system.

The Obuchowski index values of DRCDS were 0.92, 0.87, and 0.86 for the internal test set and external test set-1 and -2, which were comparable or slightly higher than those of the reports and Model-C alone (Table 3; Figure 4). After using the DRCDS, 36 of the 192 patients in the internal test set first diagnosed by Model-C were deemed unreliable by decision model and were deferred to the radiologist. Among them, 23 patients received a positive impact (consist with or closer to the reference standard). For external test set-1 (*n* = 99) and −2 (*n* = 50), the positive impact rates were 19/26 and 3/4, respectively. Compared to Model-C alone, DRCDS improved specificity by 0.00–0.04, 0.11–0.40, and 0.00–0.06 (at non-inferior sensitivity) or sensitivity by 0.04–0.19, 0.08–0.15, and 0.00–0.07 (at non-inferior specificity) for the internal test set and external test set-1 and -2, respectively. When compared to the reports, DRCDS improved specificity by 0.00–0.03, 0.00, and 0.06–0.13 (at non-inferior sensitivity) or sensitivity by 0.03–0.09, 0.00–0.04, and 0.00–0.07 (at non-inferior specificity), respectively. Representative cases are in Figure 5.

**Table 3:** The diagnostic performance of DRCDS compared to that of Standalone reports, Model-C and serum fibrosis tests

	Significant fibrosis (S2-4)	Significant fibrosis (S2-4)	Advanced fibrosis (S3-4)	Advanced fibrosis (S3-4)	Cirrhosis (S4)	Cirrhosis (S4)	Obuchowski index
	Sensitivity	Specificity	Sensitivity	Specificity	Sensitivity	Specificity	
Internal Test Set							
Reports	0.67 (0.58, 0.76) [68/101]	0.90 (0.84, 0.96) [82/91]	0.66 (0.55, 0.76) [52/79]	0.93 (0.88, 0.98) [105/113]	0.66 (0.54, 0.77) [42/64]	0.93 (0.89, 0.97) [119/128]	0.91 (0.89, 0.93)
Model-C OP1	0.69 (0.60, 0.78) [70/101]	0.93 (0.88, 0.99) [85/91]	0.65 (0.54, 0.75) [51/79]	0.92 (0.87, 0.97) [104/113]	0.67 (0.56, 0.79) [43/64]	0.89 (0.84, 0.94) [114/128]	0.90 (0.87, 0.92)
Model-C OP2	0.72 (0.64, 0.81) [73/101]	0.90 (0.84, 0.96) [82/91]	0.51 (0.40, 0.62) [40/79]	0.93 (0.88, 0.98) [105/113]	0.52 (0.39, 0.64) [33/64]	0.94 (0.90, 0.98) [120/128]	0.90 (0.87, 0.92)
DRCDS OP1	0.69 (0.60, 0.78) [70/101]	0.93 (0.88, 0.99) [85/91]	0.67 (0.57, 0.77) [53/79]	0.94 (0.89, 0.98) [106/113]	0.69 (0.57, 0.80) [44/64]	0.93 (0.89, 0.97) [119/128]	0.92 (0.90, 0.94)
DRCDS OP2	0.76 (0.68, 0.85) [77/101]	0.91 (0.85, 0.97) [83/91]	0.70 (0.59, 0.80) [55/79]	0.93 (0.88, 0.98) [105/113]	0.69 (0.57, 0.80) [44/64]	0.93 (0.89, 0.97) [119/128]	0.92 (0.90, 0.94)
APRI OP1	0.67 (0.58, 0.76) [68/101]	0.81 (0.73, 0.89) [74/91]	0.65 (0.54, 0.75) [51/79]	0.81 (0.73, 0.88) [91/113]	0.66 (0.54, 0.77) [42/64]	0.78 (0.71, 0.85) [100/128]	0.80 (0.76, 0.84)^b^
APRI OP2	0.34 (0.24, 0.43) [34/101]	0.90 (0.84, 0.96) [82/91]	0.14 (0.06, 0.22) [11/79]	0.93 (0.88, 0.98) [105/113]	0.06 (0.00, 0.12) [4/64]	0.93 (0.89, 0.97) [119/128]	0.80 (0.76, 0.84)^b^
FIB-4 OP1	0.71 (0.62, 0.80) [72/101]	0.74 (0.65, 0.83) [67/91]	0.66 (0.55, 0.76) [52/79]	0.75 (0.67, 0.83) [85/113]	0.66 (0.54, 0.77) [42/64]	0.73 (0.66, 0.81) [94/128]	0.81 (0.76, 0.85)^b^
FIB-4 OP2	0.49 (0.39, 0.58) [49/101]	0.90 (0.84, 0.96) [82/91]	0.24 (0.15, 0.33) [19/79]	0.94 (0.89, 0.98) [106/113]	0.16 (0.07, 0.25) [10/64]	0.95 (0.91, 0.98) [121/128]	0.81 (0.76, 0.85)^b^
External Test Set-1							
Reports	0.95 (0.90, 0.99) [89/94]	0.60 (0.17, 1.00) [3/5]	0.88 (0.81, 0.96) [69/78]	0.86 (0.71, 1.00) [18/21]	0.92 (0.85, 1.00) [48/52]	0.66 (0.52, 0.80) [31/47]	0.87 (0.85, 0.89)
Model-C OP1	0.97 (0.93, 1.00) [91/94]	0.20 (0.00, 0.40) [1/5]	0.87 (0.80, 0.95) [68/78]	0.67 (0.47, 0.87) [14/21]	0.92 (0.85, 1.00) [48/52]	0.55 (0.41, 0.70) [26/47]	0.85 (0.82, 0.88)
Model-C OP2	0.81 (0.73, 0.89) [76/94]	0.60 (0.17, 1.00) [3/5]	0.77 (0.68, 0.86) [60/78]	0.86 (0.71, 1.00) [18/21]	0.88 (0.80, 0.97) [46/52]	0.68 (0.55, 0.81) [32/47]	0.85 (0.82, 0.88)
DRCDS OP1	0.95 (0.90, 0.99) [89/94]	0.60 (0.17, 1.00) [3/5]	0.88 (0.81, 0.96) [69/78]	0.86 (0.71, 1.00) [18/21]	0.92 (0.85, 1.00) [48/52]	0.66 (0.52, 0.80) [31/47]	0.87 (0.85, 0.89)
DRCDS OP2	0.96 (0.92, 1.00) [90/94]	0.60 (0.17, 1.00) [3/5]	0.88 (0.81, 0.96) [69/78]	0.86 (0.71, 1.00) [18/21]	0.96 (0.91, 1.00) [50/52]	0.66 (0.52, 0.80) [31/47]	0.87 (0.85, 0.89)
APRI OP1	1.00 (1.00, 1.00) [94/94]	0.00 (0.00, 0.00) [0/5]	0.87 (0.80, 0.95) [68/78]	0.14 (0.00, 0.29) [3/21]	0.92 (0.85, 1.00) [48/52]	0.06 (0.00, 0.13) [3/47]	0.71 (0.65, 0.77)^b^
APRI OP2	0.51 (0.41, 0.61) [48/94]	0.60 (0.20, 1.00) [3/5]	0.23 (0.14, 0.32) [18/78]	0.86 (0.71, 1.00) [18/21]	0.46 (0.33, 0.60) [24/52]	0.66 (0.52, 0.80) [31/47]	0.71 (0.65, 0.77)^b^
FIB-4 OP1	0.94 (0.89, 0.99) [88/94]	0.40 (0.00, 0.80) [2/5]	0.88 (0.81, 0.96) [69/78]	0.38 (0.17, 0.59) [8/21]	0.92 (0.85, 1.00) [48/52]	0.19 (0.08, 0.30) [9/47]	0.78 (0.72, 0.84)^a^
FIB-4 OP2	0.41 (0.32, 0.51) [39/94]	0.60 (0.20, 1.00) [3/5]	0.45 (0.34, 0.56) [35/78]	0.86 (0.71, 1.00) [18/21]	0.46 (0.33, 0.60) [24/52]	0.70 (0.57, 0.83) [33/47]	0.78 (0.72, 0.84)^a^
External Test Set-2							
Reports	0.84 (0.71, 0.97) [26/31]	0.68 (0.48, 0.89) [13/19]	0.79 (0.57, 1.00) [11/14]	0.81 (0.68, 0.93) [29/36]	1.00 (1.00, 1.00) [5/5]	0.76 (0.63, 0.88) [34/45]	0.84 (0.81, 0.87)
Model-C OP1	0.84 (0.71, 0.97) [26/31]	0.68 (0.48, 0.89) [13/19]	0.79 (0.56,1.00) [11/14]	0.86 (0.75, 0.97) [31/36]	1.00 (1.00, 1.00) [5/5]	0.89 (0.80, 0.98) [40/45]	0.85 (0.82, 0.88)
Model-C OP2	0.84 (0.71, 0.97) 26/31]	0.68 (0.48, 0.89) [13/19]	0.79 (0.57, 1.00) [11/14]	0.86 (0.75, 0.97) [31/36]	1.00 (1.00, 1.00) [5/5]	0.89 (0.80, 0.98) [40/45]	0.85 (0.82, 0.88)
DRCDS OP1	0.84 (0.71, 0.97) [26/31]	0.74 (0.54, 0.93) [14/19]	0.79 (0.57, 1.00) [11/14]	0.92 (0.83, 1.00) [33/36]	1.00 (1.00, 1.00) [5/5]	0.89 (0.80, 0.98) [40/45]	0.86 (0.83, 0.89)
DRCDS OP2	0.90 (0.80, 1.00) [28/31]	0.68 (0.48, 0.89) [13/19]	0.86 (0.67, 1.00) [12/14]	0.81 (0.68, 0.93) [29/36]	1.00 (1.00, 1.00) [5/5]	0.89 (0.80, 0.98) [40/45]	0.86 (0.83, 0.89)
APRI OP1	0.83 (0.68, 0.98) [20/24]	0.69 (0.44, 0.94) [9/13]	0.80 (0.60, 1.00) [8/10]	0.52 (0.33, 0.71) [14/27]	1.00 (1.00, 1.00) [4/4]	0.49 (0.31, 0.66) [16/33]	0.72 (0.67, 0.78)^b^
APRI OP2	0.83 (0.68, 0.98) [20/24]	0.69 (0.44, 0.94) [9/13]	0.3 (0.02, 0.58) [3/10]	0.85 (0.72, 0.99) [23/27]	0.25 (0.00, 0.50) [1/4]	0.82 (0.69, 0.95) [27/33]	0.72 (0.67, 0.78)^b^
FIB-4 OP1	0.83 (0.68, 0.98) [20/24]	0.31 (0.06, 0.56) [4/13]	0.80 (0.60, 1.00) [8/10]	0.44 (0.26, 0.63) [12/27]	1.00 (1.00, 1.00) [4/4]	0.46 (0.28, 0.62) [15/33]	0.70 (0.65, 0.75)^b^
FIB-4 OP2	0.38 (0.18, 0.57) [9/24]	0.69 (0.44, 0.94) [9/13]	0.50 (0.19, 0.81) [5/10]	0.78 (0.62, 0.93) [21/27]	0.50 (0.01, 0.99) [2/4]	0.73 (0.58, 0.88) [24/33]	0.70 (0.65, 0.75)^b^

Data in parentheses are 95% confidence interval.

OP1 and OP2 refer to a set of distinct parameters that determine an operating point for each application.

APRI, Aspartate transaminase-to-platelet ratio index; DRCDS, Deep learning – radiologist Complementarity Decision System; FIB-4, Fibrosis-4 index.

a Significantly different from the results of DRCDS (*p* < 0.01). Mann-Whitney U test was performed.

b Significantly different from the results of DRCDS (*p* < 0.001). Mann-Whitney U test was performed.

In the internal test set, external test set-1 and -2, 81.3%, 73.7%, and 92.0% of cases were directly proceeded with Model-C (i.e., the decision model deemed Model-C reliable), with the AUCs for significant fibrosis, advanced fibrosis, and cirrhosis were 0.94, 0.95, and 0.94, 0.94, 0.91, and 0.83, and 0.81, 0.90, and 0.93, respectively. The Obuchowski index values of Model-C were improved to 0.92, 0.86, and 0.85, respectively. The Obuchowski index values of reports were 0.87, 0.86, and 0.84, which was inferior to Mode-C in Internal Test Set (*p* = 0.001), but the difference in external test set −1 and −2 were not significant (*p* = 0.64 for both) (Table S3).

### Comparison between DRCDS, serum markers, and TE

The results of APRI and FIB-4 were available in all patients in tnternal test set and external test set-1, and 37 patients in external test set-2. In all test sets, the AUCs of DRCDS were higher than APRI and FIB-4 in diagnosing all fibrosis stages. The Obuchowski index value of DRCDS (0.86–0.92) was also higher than that of APRI (0.71–0.80, all *p* < 0.01) and FIB-4 (0.70–0.81, *p* < 0.01 for the internal test set and external test set-2, *p* = 0.005 for external test set-1) (Table 3; Figure 4).

**Figure 6:**
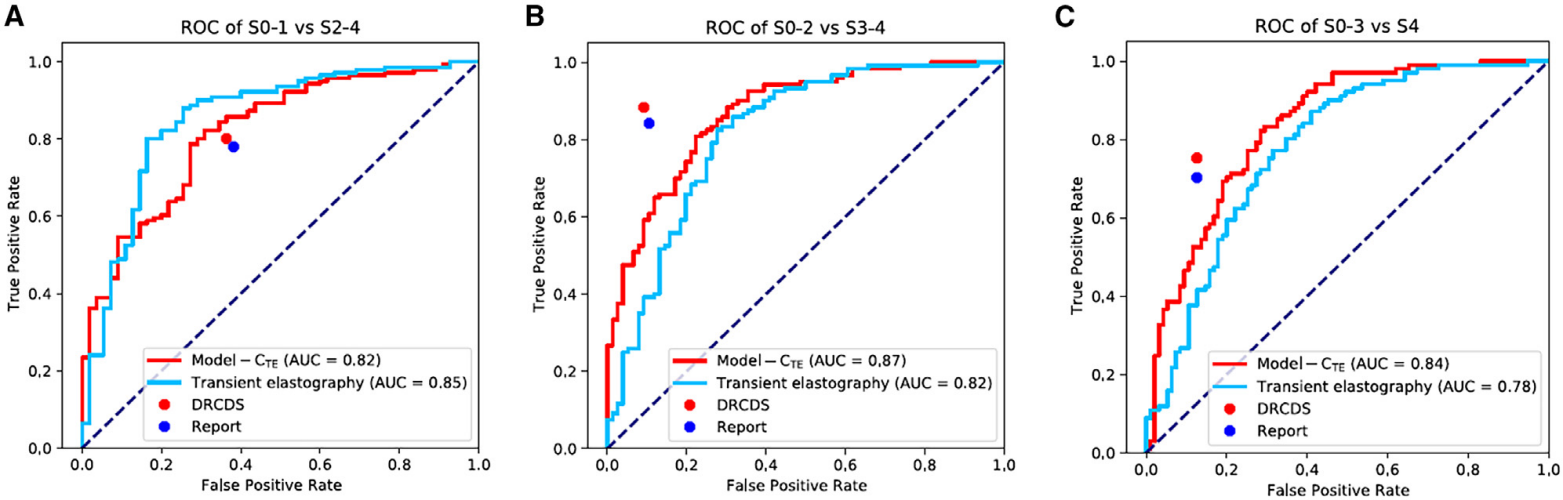
Performance of DRCDS compared to that of standalone Model-C_TE_, reports and TE on transient elastography test set DRCDS, deep learning—radiologist complementarity decision system. (A) AUC of S0-1 vs S2-4. (B) AUC of S0-2 vs S3-4. (C) AUC of S0-3 vs S4.

The difference between the Obuchowski index value of DRCDS and TE was not significant (0.90 vs. 0.89, *p* = 0.73). When diagnosing advanced fibrosis and cirrhosis, DRCDS improved specificity by 0.20 and 0.15 (at non-inferior sensitivity) or sensitivity by 0.53 and 0.37 (at non-inferior specificity) respectively, while TE performed better when diagnosing significant fibrosis. The Obuchowski index of the DRCDS was slightly higher than that of Model-C_TE_ (0.90 vs. 0.87, *p* = 0.24) and the reports (0.90 vs. 0.88, *p* = 0.49). The sensitivity and specificity were improved for all fibrosis stages compared to the reports alone and for advanced fibrosis and cirrhosis compared to Model-C_TE_ (Table 4; Figure 6). Consistent with the Internal Test Set results, Model-C_TE_ outperformed Model-L_TE_ and Model-S_TE_ (Table S4). In addition, the difference between Obuchowski index values of Model-C and Model-C_TE_ was not significant (*p* = 0.13). The equivalence testing also claimed equivalence (The greater of the two *P* is 0.00).

**Table 4:** The diagnostic performance of DRCDS compared to that of Standalone reports, Model-C_TE_ and TE on TE test set

	Significant fibrosis (S2-4)	Significant fibrosis (S2-4)	Advanced fibrosis (S3-4)	Advanced fibrosis (S3-4)	Cirrhosis (S4)	Cirrhosis (S4)	Obuchowski index
	Sensitivity	Specificity	Sensitivity	Specificity	Sensitivity	Specificity	
Reports	0.78 (0.71, 0.85) [110/141]	0.62 (0.49, 0.75) [34/55]	0.84 (0.78, 0.91) [101/120]	0.89 (0.83, 0.96) [68/76]	0.70 (0.61, 0.79) [71/101]	0.87 (0.81, 0.94) [83/95]	0.88 (0.84, 0.92)
Model-C_TE_ OP1	0.79 (0.72, 0.85) [111/141]	0.73 (0.61, 0.84) [40/55]	0.83 (0.77, 0.90) [100/120]	0.74 (0.64, 0.84) [56/76]	0.70 (0.61, 0.79) [71/101]	0.80 (0.72, 0.88) [76/95]	0.87 (0.84, 0.90)
Model-C_TE_ OP2	0.84 (0.78, 0.90) [119/141]	0.64 (0.51, 0.76) [35/55]	0.56 (0.47, 0.65) [67/120]	0.91 (0.84, 0.97) [69/76]	0.48 (0.38, 0.57) [48/101]	0.87 (0.81, 0.94) [83/95]	0.87 (0.84, 0.90)
DRCDS OP1	0.78 (0.71, 0.85) [110/141]	0.64 (0.51, 0.76) [35/55]	0.85 (0.79, 0.91) [102/120]	0.91 (0.84, 0.97) [69/76]	0.71 (0.62, 0.80) [72/101]	0.88 (0.82, 0.95) [84/95]	0.90 (0.86, 0.94)
DRCDS OP2	0.82 (0.75, 0.88) [115/141]	0.62 (0.49, 0.75) [34/55]	0.88 (0.83, 0.94) [106/120]	0.89 (0.83, 0.96) [68/76]	0.75 (0.67, 0.84) [76/101]	0.87 (0.81, 0.94) [83/95]	0.90 (0.86, 0.94)
TE OP1	0.80 (0.74, 0.87) [113/141]	0.84 (0.74, 0.93) [46/55]	0.83 (0.77, 0.90) [100/120]	0.71 (0.61, 0.81) [54/76]	0.71 (0.62, 0.80) [72/101]	0.73 (0.64, 0.82) [69/95]	0.89 (0.85, 0.93)
TE OP2	0.91 (0.86, 0.96) [128/141]	0.60 (0.47, 0.73) [33/55]	0.35 (0.26, 0.44) [42/120]	0.91 (0.84, 0.97) [69/76]	0.38 (0.28, 0.47) [38/101]	0.87 (0.81, 0.94) [83/95]	0.89 (0.85, 0.93)

Data in parentheses are 95% confidence interval.

OP1 and OP2 refer to a set of distinct parameters that determine an operating point for each application.

DRCDS, Deep learning – Radiologist Complementarity Decision System; TE, Transient elastography.

## Discussion

This study introduces a portal venous CT-based DL-radiologist complementarity decision system with essential design improvements (combining liver and spleen features), enhanced generalizability (test-time adaptation), and complementary decision-making (decision model). The DRCDS achieved satisfactory diagnostic performance in both the internal (Obuchowski index:0.92) and external (Obuchowski index:0.87 and 0.86) test sets and reduced the requirement for radiologist assessment by 73.7%–92.0%. The DRCDS also surpassed APRI and FIB-4 and performed comparable to TE.

Predictive AI tools and radiologists demonstrated complementary strengths in different clinical scenarios.^11^^,^^12^ Our research also yielded similar findings—radiologists excelled in diagnosing cirrhosis, whereas Model-C proved more effective in diagnosing early liver fibrosis. Therefore, the combination of them could enhance the efficiency and accuracy of medical diagnosis. However, effective integration of AI and human expertise remains challenging, since presenting AI results directly can introduce unconscious cognitive biases to radiologists,^13^ and senior radiologists may prioritize their own judgment.^14^^,^^15^ Moreover, under which conditions it is reliable to solely rely on AI tools is unclear. The recently proposed CoDoC system increases the credibility of AI tools and harnesses the complementary strengths of radiologists and AI tools, through using confidence scores derived from the predictive AI to assess whether to rely on the AI diagnosis or defer to radiologists.^16^ As CoDoC is primarily used for binary classifications, we improved the existing deferral AI model and introduced an ordinal encoding-based decision model to accommodate Model-C, which combines multiple binary classification tasks. Our results showed that the Obuchowski index values, sensitivity, and specificity mostly improved when using the DRCDS compared with radiologists or Model-C alone. With the oversight of decision model, the diagnostic performance of Model-C was superior to senior radiologists, showing its potential to be used alone. Furthermore, Model-C can provide a more detailed four-level classification, rather than three-level classification by radiologists in clinical practice. Thus, this system can be used to diagnose and monitor the degree of liver fibrosis at no extra examinations and cost.

Several DL-based models have been proposed for precise and reproducible liver fibrosis diagnosis.^6^^,^^7^^,^^8^^,^^9^^,^^10^^,^^17^^,^^18^^,^^19^ The DL system proposed by Choi et al.^9^ achieved an AUC of 0.95–0.97 and an Obuchowski index of 0.94, but the system did not consider portal venous pressure-related signs in liver fibrosis diagnosis. We designed a DL model that integrates information from the liver and spleen, which consists with clinicians’ diagnostic approaches and liver fibrosis pathological process. Our model combining liver and spleen outperformed the model using liver only, perhaps because spleen volume and structure consistently change as liver fibrosis progresses, even in the early stages, whereas the total liver volume is an incomplete predictor of underlying fibrosis.^20^^,^^21^^,^^22^ Moreover, spleen characteristics are less influenced by active hepatitis or steatosis, which are confounding factors if only liver region is considered.^23^ Accordingly, our stratification analysis demonstrated that inflammation did not influence the performance of Model-C, which has not been investigated in previous studies. Although the AUC and Obuchowski values of Model-C was not as high as DL system proposed by Choi et al., one of the objectives of this study was to explore the improvement by considering both liver and spleen within the same DL architecture, in contrast to just liver. Moreover, their DL system was trained using a considerably larger cohort and the patient distribution in the test sets may have influenced model’s performance.

The performance of diagnostic models can often degrade with external validation owing to the intrinsic nature of real-world medical data,^24^ especially in DL models that heavily rely on their development datasets. However, most previous studies lack of independent external validations^6^^,^^8^^,^^17^^,^^18^^,^^19^^,^^25^ or methods to mitigate domain shifts.^9^^,^^10^ To enhance our model’s generalization capability, we adopted a test-time learning algorithm to alleviate variations caused by scanner parameters, resolution, intensity, and contrast variations. This reduced errors by minimizing entropy, facilitating adaptation during testing without requiring labels from the target datasets.^26^ Test-time adaptation in medical image diagnosis is a promising approach for effectively deploying DL methods in real-world clinical practice.

We significantly reduced the time and labor required for high-quality annotations for training the segmentation model by using a human-in-the-loop strategy. The manual refinement time for liver and spleen margins gradually decreased and could be completed < 2 min in the final stages, streamlining the annotation process. What’s more, our classification model could better uncover characteristics related to ordinal classification (for example, in liver fibrosis staging, S2 is closer to S1 and S0 than to S4), outperforming traditional classification models. This is due to the fact that ordinal encoding can better utilize the inherent connections between different liver fibrosis stages, unlike one-hot encoding, where the encoding of different categories is independent and completely unrelated. We also improved the existing CoDoC system limited to binary classification. Our system is capable of easy expansion to multi-classification tasks, especially for classification models based on ordinal encoding. Moreover, in the process of diagnosing S2/S3, which is harder to discriminate, we only decide to ultimately adopt the predictive AI model’s prediction if both two of the decision models simultaneously agree that the AI model should be trusted, guaranteeing the diagnostic accuracy.

In conclusion, the DRCDS using portal venous CT improved liver fibrosis staging accuracy by improving DL model’s essential design and complementing the strengths of radiologist and AI tool. It also presented and validated solutions to issues related to big data annotation and model generalization, which are frequently encountered in prospective validation in real-world clinical practice.

### Limitations of the study

This study had several limitations. First, its retrospective nature introduced confounding factors, including missing data and selection bias. Nevertheless, our classification model upholds its performance in 3 external test sets. The subgroup analysis also showed its robust generalization. In the future, we will prospectively verify the diagnostic efficacy of DRCDS in different clinical scenarios. Second, the DL models were developed using pathological examinations as the reference standard. However, sampling errors may impact the algorithm’s effectiveness. In future studies, magnetic resonance elastography or prognosis should be used as an alternative gold standard to verify the efficacy of DRCDS. Third, the decision model based its output on the predictive AI confidence scores, without considering the radiologist’s confidence. Integrating radiologists’ diagnostic confidence into the decision model may further improve DRCDS performance. Finally, the Obuchowski index of the DRCDS did not significantly improve, perhaps owing to the small sample size of the test sets. However, it is still encouraging that the DRCDS performed comparably to the senior radiologists and TE.

## Resource availability

### Lead contact

Further information and requests for resources should be directed to and will be fulfilled by the lead contact, Huimao Zhang (huimao@jlu.edu.cn).

### Materials availability

This study did not generate new unique reagents.

### Data and code availability

Data will be made available from the lead contact on request.

All original code have been deposited at Github (https://github.com/med-air/Efficient-AI-tool-for-liver-fibrosis-staging/), and are publicly accessible as of the date of publication. DOIs are listed in the key resources table.

Any additional information required to reanalyze the data reported in this paper is available from the lead contact upon request.

## Acknowledgments

We would like to thank Editage (www.editage.cn) for English language editing.

This research was funded by National Key Research and Development Program of China (No. 2024YFE0213800). We sincerely appreciate Prof. Alfred Kow Wei Chieh from National University of Singapore for the valuable suggestions on the research direction and manuscript of this study.

## Author contributions

S.Z., W.M., M.L., L.Z., H.Z., and Q.D. designed the experiments. W.M., J.C., and Q.D. developed the model. S.Z. and W.M. created the figures. M.L. and K.H. conducted the reader study. T.Y.S., L.Z., M.L., Y.Z., F.L., S.G., L.Y., L.Z., L.W., and H.H.C.L. collected the dataset. S.Z., W.M., M.L., and W.J. performed the statistical analysis. J.N., P.JG., Q.D., and H.Z. supervised the work. S.Z. and W.M. wrote the manuscript. J.C., T.Y.S., J.N., P.G., Q.D., and H.Z. revised the paper. All authors read and approved the final version of the article.

## Declaration of interests

The authors declare no competing interests.

## STAR★Methods

### Key resources table

**Table:** 

REAGENT or RESOURCE	SOURCE	IDENTIFIER
**Deposited data**		

DataSet	This paper	N/A
Source Code	This paper	https://github.com/med-air/Efficient-AI-tool-for-liver-fibrosis-staging/

**Software and algorithms**		

Python (version 3.6.0)	Python software	https://www.python.org/
MedCalc (version 16.8)	MedCalc Software	https://www.medcalc.org/
Minitab (version 18.0)	Minitab Statistical Software	https://www.minitab.com/zh-cn/

### Experimental model and study participant details

#### Study population

In this multi-center retrospective study, Institutional Review Board approval was obtained from The First Hospital of Jilin University, The First Hospital of Lanzhou University, Sichuan Academy of Medical Sciences & Sichuan Provincial People’s Hospital and Prince of Wales Hospital. The requirement for written informed consent was waived.

We used a multi-center dataset comprising 994 consecutive patients between June 2010 and January 2022 from four hospitals across China. We included patients aged ≥18 years who underwent portal venous phase abdominal CT examination and histopathological liver fibrosis staging evaluation <6 months post-CT. Patients with a history of previous liver or splenic surgery, hepatic tumors ≥5cm or located on the edge causing liver contour alteration, and poor image quality were excluded. Clinical data, laboratory findings, and pathological examinations were obtained from medical records. Patients enrolled between January 2015–2020 in Changchun were randomly allocated to the training (n=502) and validation sets (n=125) at a ratio of 8:2, while those enrolled between January 2020–2022 were allocated to the internal test set (n=192). Patients from the other three hospitals constituted the external test sets (n=99, 50 and 26).

### Method details

#### Imaging protocol

CT data obtained in the axial plane were collected from different manufacturers (Siemens, GE Healthcare, Phillips, Neusoft, and TOSHIBA). Detailed CT imaging parameters are listed in Table S5. Portal venous phase CT images were acquired 70–80 s after intravenous contrast agent administration.

#### Reference standards

Liver fibrosis staging was determined through pathological examination of liver specimens obtained via liver resection or US-guided percutaneous liver biopsy, based on the Scheuer grading and staging system: S0 (none), S1 (enlarged fibrotic portal tracts), S2 (periportal or portal-portal septa with intact architecture), S3 (fibrosis with architectural distortion but no obvious cirrhosis), and S4 (probable or definite cirrhosis).^27^

#### DRCDS development

The DRCDS consisted of three sequential modules: a liver and spleen segmentation model, liver fibrosis classification model and a decision model:

(i) Segmentation model: We used TransUNet^28^ for backbone of liver and spleen segmentation because it can balance both efficiency and segmentation performance (Figure S2). Specifically, the images were first resampled to size of 224 × 224 in transverse plane. The image HU values were truncated to the range of [-200, 250] and were further normalized to zero-mean and unit variance. The hyperparameters used in this segmentation model were the same with TransUNet. During this process, an iterative human-in-the-loop strategy^29^ was used to minimize annotation time (Figure S3). Specifically, using a small-scale labelled public dataset,^30^ we first trained the lightweight segmentation network that can yield a reasonable performance relying on our previous work.^31^ The coarse annotations of unlabeled internal set were generated by the trained lightweight network and then refined by a radiologist (with 6 years of experience). The radiologist was advised to select cases for refinement in an easy-to-hard manner to minimize the time for manual annotation. The refined annotations were next used to train the TransUNet model. The whole process was repeated several times. All the 819 cases from internal set and 175 cases from external sets were labeled in this way.
(ii) Classification model: The segmented liver and spleen regions were used as input and processed by ResNet-50^32^ for feature extraction and subsequent long short-term memory module^33^ processing to capture three-dimensional spatial relationships (Figure S4). Rather than using conventional one-hot labels, labels were converted into 4-bit ordinal vectors for binary-class classification tasks (S0 vs. S1–4, S0–1 vs. S2–4, S0–2 vs. S3–4, S0–3 vs. S4). This conversion was carried out because it was capable of capitalizing on the inherent relationships between different stages. The final prediction is determined by the highest bit exceeding a cut-off value (i.e., 0.5 in our study). During the training phase, we used the Adam optimizer with an initial learning rate of 1e-5 and a weight decay of 1e-4. The learning rate was halved every 10 epochs while the model was trained for 30 epochs in total.

In addition, test-time adaptation using the entropy minimization method was used to address data distribution shifts in external sets.^34^ At model inference, the network was quickly updated on the test data without requiring the labels of liver fibrosis stages. In particular, the parameters of BN layers were updated via self-supervised learning while other parameters in the network being fixed. The optimization objective was minimizing Shannon entropy of the model prediction *P*, which can be calculated by Entropy (*P*) = PlogP.

Besides Model-C (liver and spleen), Model-L (only liver) and Model-S (only spleen) were also developed and their performance was compared. Moreover, the performance of Model-C was evaluated by stratification according to patient characteristics, pathology, and CT acquisition data.

(iii) Decision model: We extended the deferral AI in CoDoC system^16^ for binary classification and introduced an ordinal encoding-based decision model tailored to our approach (Figure S5). Our proposed decision model utilizes the confidence score generated by Model-C to determine whether to trust the output of Model-C or defer the case to radiologists. Model-C generates 4-bit ordinal vectors (i.e., 4 values ranging from 0 to 1, representing the probability of different sub-binary classification tasks) to predict the final liver fibrosis stage, which is called ordinal encoding strategy. In this process, we train three sub-decision models: $D_{2-4}$, $D_{3-4}$ and $D_{4}$, which handle the three sub-binary classification tasks: S0-1 vs S2-4, S0-2 vs S3-4, and S0-3 vs S4, respectively. During the inference phase, if the model's output $\hat{y}$ is S0-1, we input the probability of S2-4 into the $D_{2-4}$ model to determine whether to trust the output. If $\hat{y}$ is S4, we input the probability of S4 into the $D_{4}$ model for evaluation. If the output $\hat{y}$ is S2/S3, we input the probabilities of (S2-4, S3-4) / (S3-4, S4) into the ($D_{2-4}$, $D_{3-4}$) / ($D_{3-4}$, $D_{4}$) models, for assessment respectively. In this case, we only decide to ultimately adopt the predictive AI model's prediction if both two of the mentioned deferral AI models simultaneously agree that the AI model should be trusted. Otherwise, we will defer to the expert.

For the training of the decision model, we take $D_{2-4}$ as an example. It uses the probability value for distinguishing between S0-1 and S2-4 as the confidence score. Here, we use z to represent this probability value. We aim to train the decision model to optimize the objective:

λ·Sensitivity+(1−λ)·Specificity,

where λϵ[0,1] is a trade-off parameter. The objective can be optimized by first computing the advantage function:

$$Advantage\;\left(z\right)=\left\{ \begin{array}{c} \left(1-\lambda \right)p\left(z,h=0|y=0\right)-\lambda p\left(z,h=0|y=1\right)\;\theta <z\\ -\left(\left(1-\lambda \right)p\left(z,h=1|y=0\right)-\lambda p\left(z,h=1|y=1\right)\right)\;z\leq \theta \end{array}\right.\text{,}$$

where h denotes the opinion of radiologist and y denotes the ground truth. θ is the threshold used to determine whether a case is considered positive or negative. Next, we determine whether to trust the results of the predictive AI model based on the value of Advantage (z): If Advantage (z) ≥ 0, we defer the case to a radiologist for diagnosis; otherwise, we directly use the results of the predictive AI model. Therefore, the key point of training the decision model lies in estimating p(z,h=a|y=b), a,b∈{0,1}. This conditional density can be factored as p(z,h=a|y=b) = p(z|h=a,y=b)·p(h=a|y=b). For p(h=a|y=b), it can be obtained from historical data. For p(z|h=a,y=b), we use a discretized kernel density estimation method^35^ for estimation. Finally, we use the validation set of the predictive AI model as the tuning set for the deferral AI model. During this process, we freeze the parameters of the predictive AI model and adjust the hyperparameters in the decision model using a grid-based hyperparameter search to obtain the optimal decision model. The hyperparameters include the operating point θ, trade-off parameter λ and the hyperparameters in kernel density estimation method (i.e., pseudocounts κ, number of bins T and smoothing bandwidth σ).

Computer codes are available online at: https://github.com/med-air/Efficient-AI-tool-for-liver-fibrosis-staging/.

#### Conventional noninvasive tools

(i) Radiologist assessment: The radiologist assessment was extracted from the radiology reports. In the included institutions, the diagnostic reports were interpreted by both a junior and a senior radiologist with <10 and >10 years of experience each and it is routine for radiologists to suggest basically normal (S0–1), chronic liver disease (S2–3) and cirrhosis (S4) on the basis of typical imaging findings.^9^ To achieve the four-level classification, two senior radiologists (L.M. and K.H., with 12 and 15 years of experience respectively) reclassified the cases diagnosed as S2–3 into S2 or S3. Any disagreements were adjudicated by a third reader.
(ii) Serum markers: APRI and FIB-4 were calculated using laboratory test results performed <7 days from the liver pathologic examination. Calculation equations are as follows: APRI = AST(U/L)/(PLT($10^{9}$/L)), FIB-4 = Age(years) × AST(U/L)/ (PLT($10^{9}$/L) ×√ALT (U/L)).
(iii) Transient elastography: TE examination was performed by a sonographer (8 years of experience; FibroTouch-FT-B; Wuxi Hisky Medical Technologies, China) <3 months before the liver pathologic examination. Owing to the limited number of patients who underwent TE in the internal set, a new allocation for TE examination was performed, which involved the retraining of the DL classification models (Model-C_TE_, Model-L_TE_, and Model-S_TE_) and the ordinal encoding-based decision model. A breakdown of the training, validation, and test sets is shown in Figure S6. Table S6 provided the characteristics of the training, validation and Transient Elastography test set.

### Quantification and statistical analysis

Continuous variables were expressed as mean ± standard deviations while categorical variables were presented as numbers (%). The DL model and NITs diagnostic performances were evaluated using the area under the receiver operating characteristic curve (AUC) and the Obuchowski index, a multinomial version of receiver operating characteristic curve analysis adapted for ordinal ref.^36^ The AUC values were compared using the DeLong et al.’s method.^37^ The Obuchowski index values were compared by using the Mann-Whitney U test and one-way ANOVA and equivalence testing.^38^ Sensitivity and specificity of NITs were reported. All analyses were conducted using Python 3.6, MedCalc 16.8 (MedCalc Software) and Minitab 18.0 (Minitab Statistical Software). *P*<0.05 was considered significant.
