# Supplementary material for: CT-based artificial intelligence system complementing deep learning model and radiologist for liver fibrosis staging
Source: iScience. 2025 Mar 17;28(4):112224. doi: 10.1016/j.isci.2025.112224 (PMC12005311; doi:10.1016/j.isci.2025.112224)
Supplement: Document S1. Figures S1–S6 and Tables S1–S6 [file mmc1.pdf]

iScience, Volume *28*

Supplemental information

CT-based artificial intelligence system complementing deep learning model and radiologist for liver fibrosis staging

Shuang Zheng, Wenao Ma, Lin Mu, Kan He, Jianfeng Cao, Tiffany Y. So, Lei Zhang, Mingyang Li, Yanan Zhai, Feng Liu, Shunlin Guo, Longlin Yin, Liming Zhao, Lei Wang, Heather H.C. Lee, Wei Jiang, Junqi Niu, Pujun Gao, Qi Dou, and Huimao Zhang

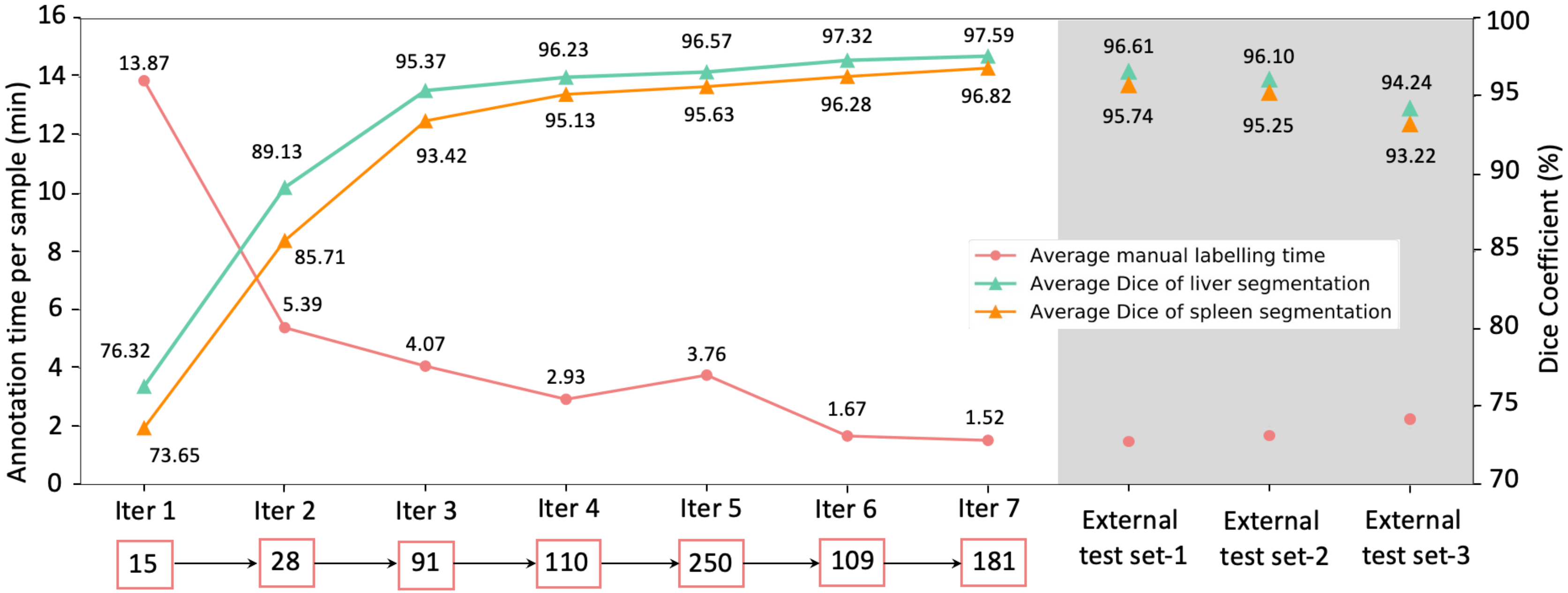

Fig. S1: The evaluation of segmentation performance together with manual labeling time with the efficient human-in-the-loop strategy, related to Results. The segmentation model was highly accurate and generalizable to external test sets after 7 iterations trained on 784 cases of internal set. Dice results for liver and spleen segmentation achieved 97.59% and 96.82% (evaluated on remaining 35 cases of Internal Test Set), 96.61% and 95.74% (External Test Set-1), 96.10% and 95.25% (External Test Set-2) and 93.22% and 94.24% (External Test Set-3). By using such human-in-the-loop strategy, the average manual refinement time of the liver and spleen segmentation decreased from 13.87 min to 1.52 min after 7 iterations.

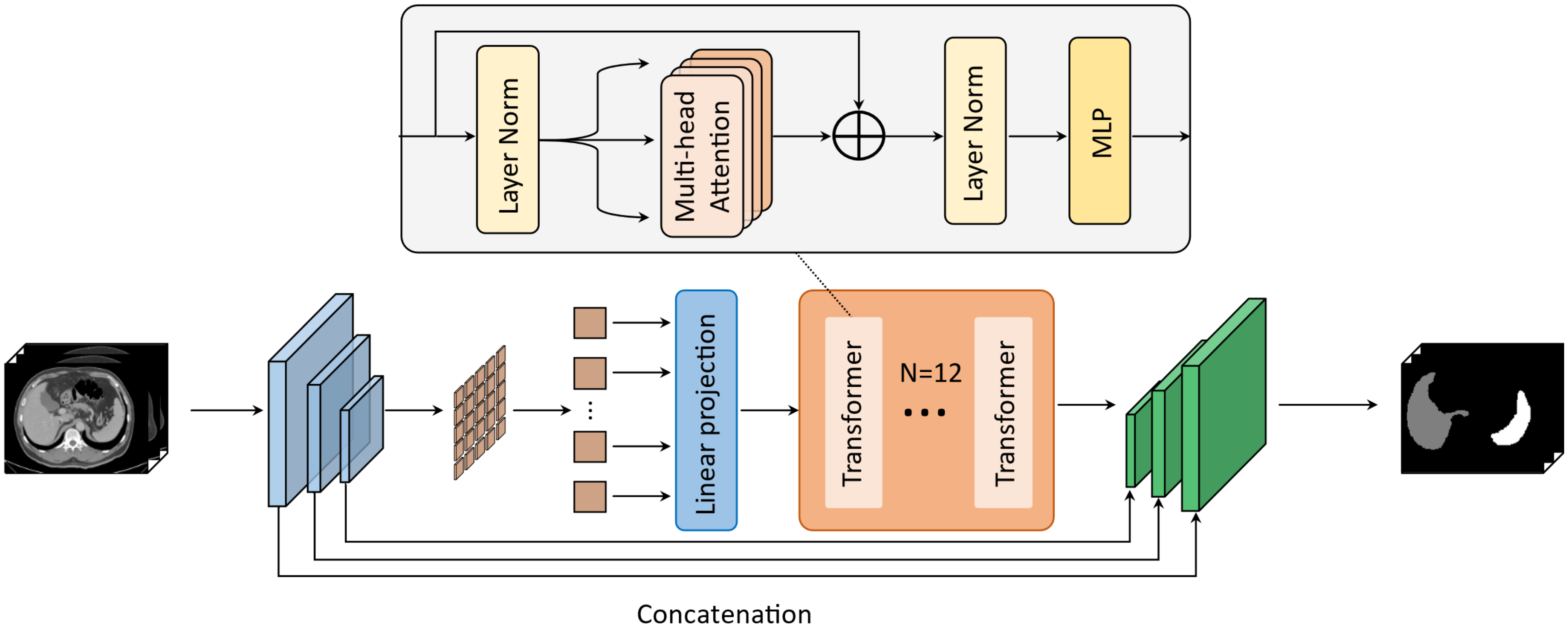

Fig. S2: The model architecture for liver and spleen multi-organ segmentation, related to STAR Methods.

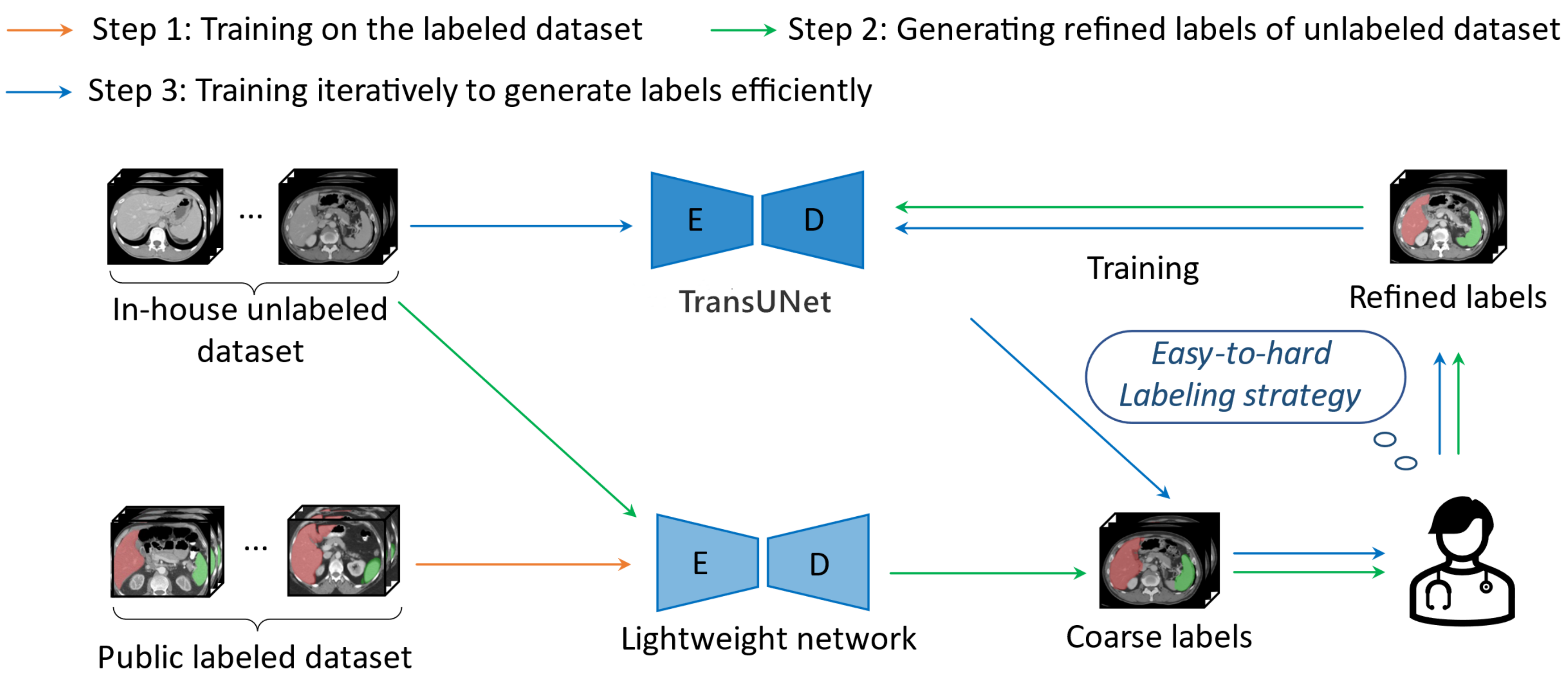

Fig. S3: Illustration of human-in-the-loop strategy for efficient annotation of segmentation labels, related to STAR Methods.

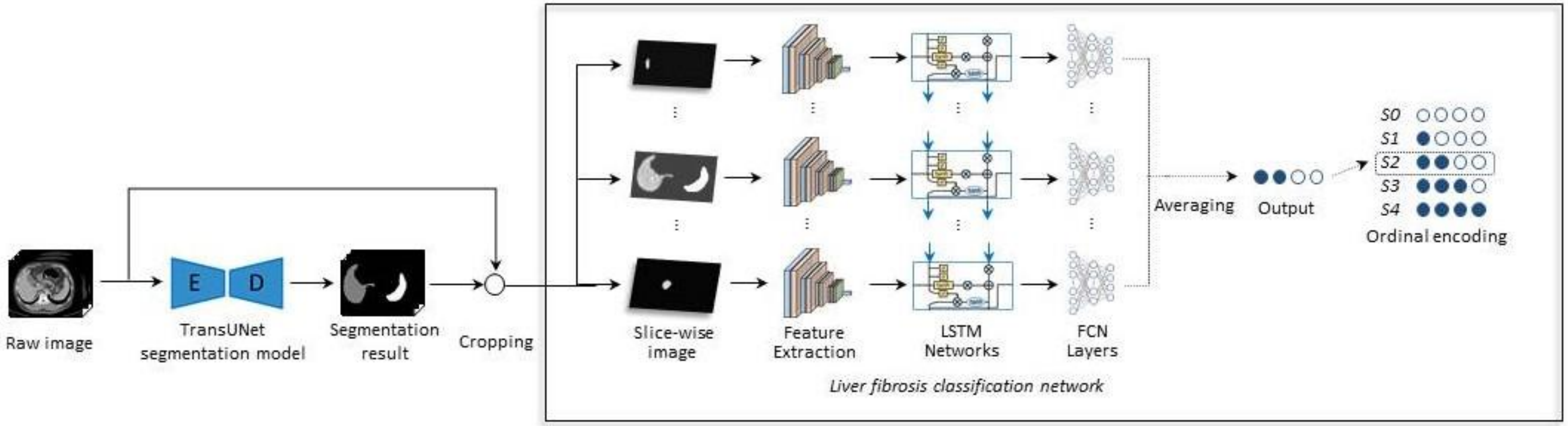

Fig. S4: The model architecture for liver fibrosis classification, related to STAR Methods.

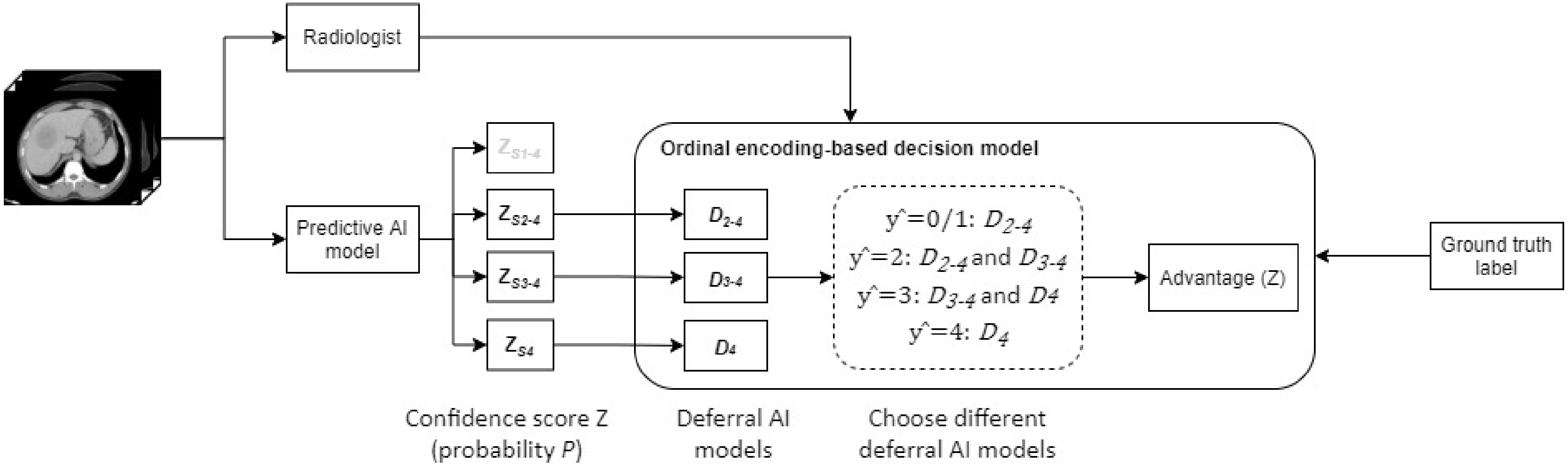

Fig. S5: Illustration of the ordinal encoding-based decision model, related to STAR Methods.

The confidence scores of predictive AI model, radiologist opinions and ground truth labels are collected to train the decision AI model to decide when to defer to the diagnosis of radiologist, and, if not deferring, to adopt the predictive AI model's prediction. The confidence score of each binary classification is forwarded into corresponding deferral AI model. We then choose different deferral AI model to compute the value of Advantage (z) based on the model prediction $\hat{y}$. If Advantage (z)$\geq$ 0, the system decides to defer to the radiologist. Otherwise, the decision model decides to use the output of the predictive AI model.

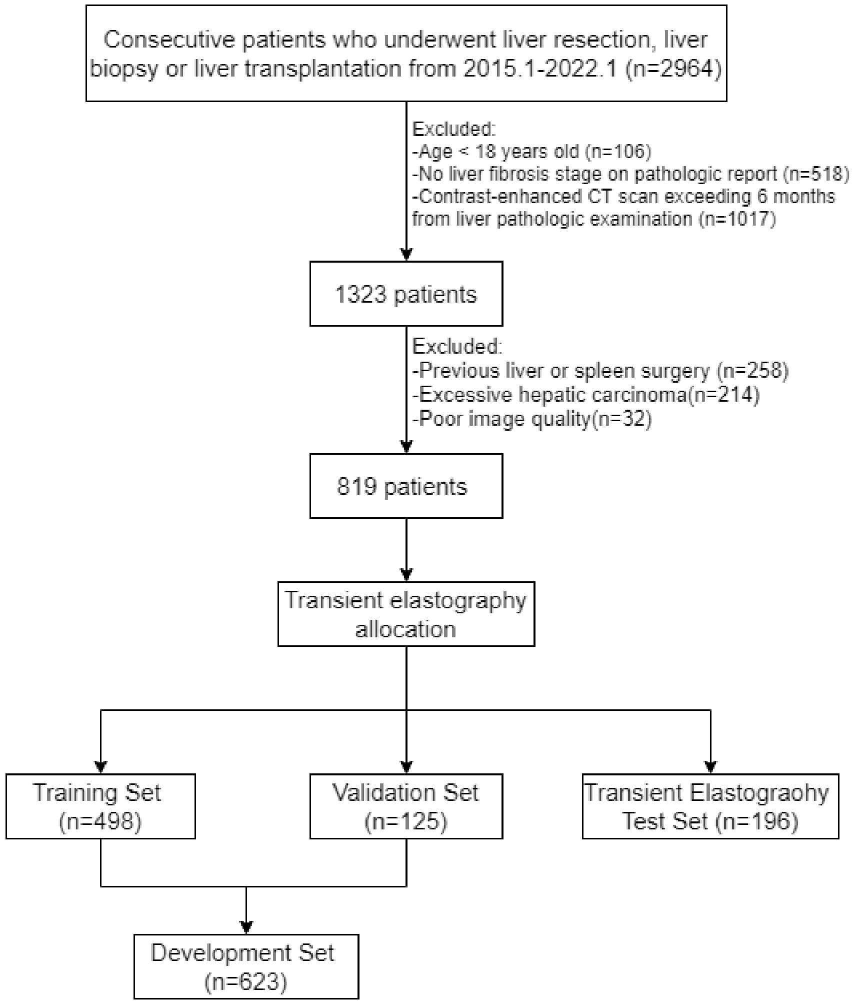

Fig. S6: Flowchart for Patient Selection in Transient Elastrography Allocation and Breakdown of Training, Validation, and Test Sets, related to STAR Methods.

Table S1 Diagnostic Performance of Model-L, Model-S and Model-C for Liver Fibrosis Staging on Internal Test Set, related to Results

	Sensitivity	Specificity	AUC
**Significant fibrosis (S2-4)**			
Model-L	0.84 (0.77, 0.91) [85/101]	0.76 (0.68, 0.86) [69/91]	0.90 (0.86, 0.94)
Model-S	0.88 (0.82, 0.94) [89/101]	0.21 (0.13, 0.29) [19/91]	0.69[†] (0.62, 0.77)
Model-C	0.83 (0.76, 0.91) [84/101]	0.82 (0.75, 0.90) [75/91]	0.92 (0.88,0.96)
**Advanced fibrosis (S3-4)**			
Model-L	0.82 (0.73, 0.90) [65/79]	0.72 (0.63, 0.80) [81/113]	0.86[*] (0.81, 0.91)
Model-S	0.87 (0.79, 0.94) [69/79]	0.28 (0.20, 0.37) [32/113]	0.70[†] (0.62, 0.78)
Model-C	0.86 (0.78, 0.94) [68/79]	0.80 (0.72, 0.87) [90/113]	0.91 (0.87, 0.95)
**Cirrhosis (S4)**			
Model-L	0.81 (0.71, 0.90) [52/64]	0.70 (0.62, 0.78) [90/128]	0.84[*] (0.78, 0.90)
Model-S	0.84 (0.75, 0.92) [55/64]	0.31 (0.23, 0.39) [40/128]	0.70[†] (0.61, 0.78)
Model-C	0.83 (0.74, 0.92) [53/64]	0.78 (0.71, 0.85) [100/128]	0.89 (0.85, 0.94)

Data in parentheses are 95% confidence interval.

AUC = area under the receiver operating characteristic curve

[*] Significantly different from the results of Model-C ($P<0.05$). DeLong's test was performed.

[†] Significantly different from the results of Model-C ($P<0.001$). DeLong's test was performed.

Table S2 Influence of Patient Characteristics, Pathology and CT Data on the Performance of Model-C for Liver Fibrosis Staging on Internal Test Set, related to Results

Characteristic	Obuchowski index	*P*
Age		0.23†
18-50y	0.92 (0.91, 0.94)	
>50y	0.89 (0.86, 0.93)	
Sex		0.77†
Women	0.90 (0.87, 0.93)	
Men	0.91 (0.88, 0.94)	
Etiologic cause		0.30†
Viral hepatitis	0.89 (0.85, 0.94)	
Others	0.92 (0.90, 0.95)	
Hepatic tumor		0.83†
Absent	0.91 (0.89, 0.94)	
Present	0.90 (0.88, 0.92)	
Pathologic specimen		0.87†
Biopsy	0.90 (0.86, 0.95)	
Resection	0.90 (0.89, 0.93)	
Liver inflammation		0.52†
G 0-2	0.92 (0.89, 0.94)	
G 3-4	0.90 (0.84, 0.95)	
CT tube voltage		0.51†
100 kVp	0.90 (0.87, 0.93)	
120 kVp	0.91 (0.88, 0.94)	
Manufacture		0.19‡
Philips	0.90 (0.87, 0.93)	
Neusoft	0.90 (0.86, 0.95)	
GE	0.89 (0.80, 0.98)	
Siemens	0.90 (0.82, 0.99)	

Data in parenthesis are 95% confidence intervals.

† Mann-Whitney U test was performed.

‡One-way ANOVA test was performed.

Table S3 Comparison of Diagnostic Performance between Model-C before and after excluding the Cases deemed unreliable by the Decision Model, related to Results

	AUC			Obuchowski index
	Significant fibrosis (S2-4)	Advanced fibrosis (S3-4)	Cirrhosis (S4)	
**Internal Test Set**				
Model-C	0.92 (0.88, 0.96)	0.91 (0.87, 0.95)	0.89 (0.85, 0.94)	0.90 (0.87, 0.92)
Model-C excluding cases deferred to radiologist	0.94 (0.89, 0.97)	0.95 (0.91, 0.98)	0.94 (0.89, 0.97)	0.92 (0.90, 0.93)
Report excluding cases deferred to radiologist	—	—	—	0.87 (0.84, 0.89)
**External Test Set-1**				
Model-C	0.86 (0.77, 0.94)	0.88 (0.81, 0.96)	0.84 (0.76, 0.92)	0.85 (0.82, 0.88)
Model-C excluding cases deferred to radiologist	0.94 (0.85, 0.98)	0.91 (0.82, 0.96)	0.83 (0.72, 0.91)	0.86 (0.83, 0.89)
Report excluding cases deferred to radiologist	—	—	—	0.86 (0.83, 0.89)
**External Test Set-2**				
Model-C	0.79 (0.68, 0.88)	0.90 (0.83, 0.97)	0.92 (0.85, 0.99)	0.85 (0.82, 0.88)
Model-C excluding cases deferred to radiologist	0.81 (0.66, 0.91)	0.90 (0.78, 0.97)	0.93 (0.81, 0.98)	0.85 (0.82, 0.88)
Report excluding cases deferred to radiologist	—	—	—	0.84 (0.81, 0.87)

Table S4 Diagnostic Performance of Model-L_{TE}, Model-S_{TE} and Model-C_{TE} for Liver Fibrosis Staging on Transient Elastography Test Set, related to Results

	Sensitivity	Specificity	AUC
**Significant fibrosis (S2-4)**			
Model-L_{TE}	0.85 (0.86, 0.96) [120/141]	0.51 (0.38, 0.61) [28/55]	0.79 (0.71, 0.87)
Model-S_{TE}	0.88 (0.82, 0.94) [124/141]	0.21 (0.13, 0.29) [11/55]	0.64† (0.55, 0.73)
Model-C_{TE}	0.89 (0.84, 0.94) [126/141]	0.53 (0.40, 0.66) [29/55]	0.82 (0.75,0.88)
**Advanced fibrosis (S3-4)**			
Model-L_{TE}	0.83 (0.78, 0.88) [100/120]	0.64 (0.54, 0.74) [50/76]	0.82* (0.76, 0.88)
Model-S_{TE}	0.88 (0.80, 0.95) [106/120]	0.28 (0.20, 0.37) [21/76]	0.65† (0.57, 0.73)
Model-C_{TE}	0.90 (0.85, 0.95) [108/120]	0.67 (0.57, 0.78) [51/76]	0.87 (0.82, 0.92)
**Cirrhosis (S4)**			
Model-L_{TE}	0.78 (0.72, 0.85) [79/101]	0.63 (0.54, 0.73) [60/95]	0.76* (0.69, 0.83)
Model-S_{TE}	0.74 (0.65, 0.8) [75/101]	0.39 (0.29, 0.49) [37/95]	0.60† (0.52, 0.68)
Model-C_{TE}	0.86 (0.79, 0.93) [87/101]	0.66 (0.57, 0.76) [63/95]	0.84 (0.78, 0.89)

Data in parentheses are 95% confidence interval

Model-L_{TE} = Model-L trained on transient elastography allocation, Model-S_{TE} = Model-S trained on transient elastography allocation, Model-C_{TE} =Model-C trained on transient elastography allocation, AUC = area under the receiver operating characteristic curve

*Significantly different from the results of Model-C_{TE} ($P<0.05$). DeLong's test was performed.

†Significantly different from the results of Model-C_{TE} ($P<0.001$). DeLong's test was performed.

Table S5 CT Imaging Techniques used for the Development and Test Sets, related to STAR Methods

CT techniques	Development Set	Internal Test Set	External Test Set-1	External Test Set-2	External Test Set-3
**Number**	627	192	99	50	26
**Tube voltage/No. (%)**					
100 kVp	79(12.6)	94(49.0)	45(45.5)	0(0.0)	4(15.4)
110 kVp	0(0.0)	0(0.0)	0(0.0)	3(6.0)	0(0.0)
120 kVp	547(87.2)	98(51.0)	52(52.5)	22(44.0)	22(84.6)
130 kVp	0(0.0)	0(0.0)	2(2.0)	25(50.0)	0(0.0)
140 kVp	1(0.2)	0(0.0)	0(0.0)	0(0.0)	0(0.0)
**Slicer thickness/No. (%)**					
5 mm	627(100.0)	192(100.0)	0(0.0)	0(0.0)	26(100.0)
8 mm	0(0.0)	0(0.0)	76(76.8)	50(100.0)	0(0.0)
10 mm	0(0.0)	0(0.0)	23(23.2)	0(0.0)	0(0.0)
**CT vendors/No. (%)**					
Siemens	120(19.1)	9(4.7)	76(76.8)	50(100.0)	5(19.2)
GE	67(10.7)	19(9.9)	0(0.0)	0(0.0)	19(73.1)
Phillips	394(62.8)	112(58.3)	22(22.2)	0(0.0)	1(3.8)
Neusoft	46(7.3)	52(27.1)	0(0.0)	0(0.0)	0(0.0)
TOSHIBA	0(0.0)	0(0.0)	0(0.0)	0(0.0)	1(3.8)

Table S6 Patient Characteristics of the Training, Validation and Transient Elastography Test Set, related to STAR Methods

Characteristic	Training Set	Validation Set	Transient Elastography Test Set
No. of patients	498	125	196
No. of men (%)	324(65.1)	84(67.2)	139(70.9)
Age(y) /mean ±SD	53.1±11.4	53.4±12.5	53.4±11.0
Underlying liver disease/No. (%)			
Hepatitis B	260(52.2)	66(52.8)	118(60.2)
Hepatitis C	45(9.0)	10(8.0)	17(8.7)
Autoimmune*	16(3.2)	4(3.2)	7(3.6)
Others†	67(13.5)	17(13.6)	44(22.4)
None	110(22.1)	28(22.4)	10(5.1)
Hepatic tumor/No. (%)			
Hepatocellular carcinoma	310(62.2)	79(63.2)	146(74.5)
Metastatic tumors	20(4.0)	6(4.8)	2(1.0)
Benign tumors‡	58(11.6)	16(12.8)	2(1.0)
None	110(22.1)	24(19.2)	46(23.5)
Pathologic confirmation/No. (%)			
US-guided biopsy	78(15.7)	21(16.8)	41(20.9)
Resection	420(84.3)	104(83.2)	155(79.1)
Histologic grade/No. (%)			
S0	129(25.9)	32(25.6)	29(14.8)
S1	62(12.4)	15(12.0)	26(13.3)
S2	60(12.0)	16(12.8)	21(10.7)
S3	28(5.6)	6(4.8)	19(9.7)
S4	219(44.0)	56(44.8)	101(51.5)

*Including autoimmune hepatitis, autoimmune cholangitis, primary biliary cirrhosis and primary sclerosing cholangitis.

†Including fatty liver disease, Wilson disease, toxic hepatitis and unknown liver disease.

‡Including cavernous hemangioma, hepatic adenoma and focal nodular hyperplasia.
